# The Influence of 25‐ and 90‐Min Afternoon Nap Opportunities on Subsequent Nighttime Sleep in Student Athletes

**DOI:** 10.1111/jsr.70120

**Published:** 2025-06-19

**Authors:** Omar Boukhris, Haresh Suppiah, Matthew Driller

**Affiliations:** ^1^ Sport, Performance, and Nutrition Research Group School of Allied Health, Human Services, and Sport, La Trobe University Melbourne Victoria Australia; ^2^ SIESTA Research Group School of Allied Health, Human Services and Sport, La Trobe University Melbourne Victoria Australia

**Keywords:** circadian rhythm, recovery, siesta, sleep architecture, sport

## Abstract

While daytime napping could support recovery in athletes, poorly timed or prolonged naps may interfere with nighttime sleep. Therefore, this study aimed to explore the effect of two different, well‐timed nap opportunity durations, 25 and 90 min, on subsequent objectively measured nighttime sleep in student athletes. Fourteen student athletes (7 females, 7 males; age: 23 ± 2 years) completed three in‐lab conditions using a randomised, counterbalanced, crossover design: no nap (NN), a 25‐min nap opportunity (N25, 14:35–15:00) and a 90‐min nap opportunity (N90, 13:30–15:00). Nighttime sleep following each condition was assessed at home via wrist actigraphy measuring bedtime, wake time, total sleep time, time in bed, wake after sleep onset (WASO) and sleep stages. Neither N25 nor N90 opportunity negatively affected the following night's sleep compared to the NN condition, including bedtime, wake time, WASO, total sleep time, light sleep, deep sleep and rapid eye movement (REM) sleep (*p* > 0.05 for all). Mean total sleep time was 7 h 13 min after NN, 7 h 5 min after N25 and 7 h 1 min after N90. In student athletes, afternoon nap opportunities of either 25 or 90 min, when ending at 15:00, did not disrupt subsequent nighttime sleep. These findings suggest that a strategically timed early afternoon nap may be a viable recovery option for this population. While individual differences in sleep need and academic demands should be considered, these results highlight the potential of well‐timed naps to support athlete recovery. Future research is needed to determine whether these effects generalise to other populations or nap timings.

## 1 Introduction

Student athletes often struggle to balance demanding training schedules with academic, social and personal responsibilities, placing them at risk for sleep loss or deprivation (Hamlin et al. 2021). Inadequate sleep among this population has been linked to impaired recovery, increased injury risk, poorer academic performance, weakened immunity and mood disturbances (Charest and Grandner 2022; Driller et al. 2022; Grandner et al. 2021; Walsh et al. 2021). Studies suggest that student athletes may experience poorer sleep quality than their non‐athlete peers (Driller, Dixon, et al. 2017), with those regularly sleep less than 8 h a night are more likely to sustain an injury (Hamlin et al. 2021).

Napping is a common strategy used by athletes, including student athletes, to enhance recovery, alleviate fatigue and optimise performance (Botonis et al. 2021; Boukhris et al. 2024; Lastella et al. 2021; Mesas et al. 2023). Between 72% and 80% of student athletes nap regularly (Mah et al. 2018; Stephenson et al. 2022), while 54% of non‐athlete university students nap at least 1–2 times per week, occurring spontaneously during the post‐lunch dip period and for durations exceeding 30 min (Lovato et al. 2014). Although the acute benefits of napping are well‐established, its impact on nighttime sleep, particularly, in student athletes, remains unclear.

The regulation of sleep is commonly explained by the two‐process model, which involves the homeostatic sleep drive (Process S), a pressure that builds with time awake and dissipates during sleep and the circadian rhythm (Process C), which directs the timing of sleep and wakefulness (Borbély 1982; Daan et al. 1984). Daytime napping can reduce homeostatic sleep pressure, potentially influencing the timing and quality of subsequent nighttime sleep (Dinges et al. 1985). For student athletes, whose schedules often disrupt regular circadian rhythms, considering how nap duration may influence these sleep‐regulation processes is important for supporting both daytime recovery and nighttime sleep.

The duration and timing of a nap play a crucial role in determining its effects on sleep architecture and overall sleep quality. Nap opportunities between 25 and 90 min, typically taken during the early afternoon (13:00–16:00), are often recommended for athletes to enhance recovery and performance (Lastella et al. 2021). This window coincides with the ‘post‐lunch dip’ in alertness, which is thought to reflect a natural circadian decline rather than simply the effect of food intake (Monk 2005). However, it is important to distinguish between fixed clock time, time since waking and an individual's circadian phase, as these factors are conceptually distinct and may influence nap effectiveness and its impact on nighttime sleep (Åkerstedt and Gillberg 1981).

Research in non‐athlete populations has shown mixed effects of napping on nighttime sleep. While some studies linked frequent (≥ 3 naps/week), long (≥ 2 h) or late naps (6:00–9:00 p.m.) to poorer nighttime sleep quality in college students (Ye et al. 2015) and older adults (Monk et al. 2001; Yoon et al. 2003), others reported no significant impact in healthy young and middle‐aged adults (Pilcher et al. 2001). These discrepancies may reflect differences in participant age, habitual napping and sleep measurement methods. Importantly, such findings may not generalise to student athletes, whose training schedules and recovery needs differ from those of the general population.

Longer naps potentially offer greater physical and cognitive benefits (Boukhris et al. 2020, 2023). However, longer nap opportunities (> 60 min) could allow for entry into deep sleep stages, potentially increasing the risk of subsequent nighttime sleep disturbances. This aspect has not been studied in student athletes, who need both daytime recovery and quality nighttime sleep to maintain their physical and academic performance and overall health. Therefore, the purpose of this study was to compare 25‐ and 90‐min afternoon nap opportunities to examine how nap duration affects nighttime sleep, including bedtime, wake after sleep onset (WASO), total sleep time and sleep stages (i.e., light, deep and REM sleep) in a cohort of student athletes.

## 2 Methods

### 2.1 Participants

The current study included 14 participants (7 females, 7 males; age: 23 ± 2 years, height: 169 ± 9 cm, body mass: 66 ± 10 kg), all of whom were university students enrolled in undergraduate or postgraduate programmes. In this study, the term student athletes refers to individuals who concurrently engage in higher education while participating in structured athletic training. According to the framework proposed by McKay et al. (2022), our participants were classified as trained/developmental individuals (Tier 2). All participants engaged in sports training four times per week on average, with a mean training duration of 8 ± 2 h weekly.

To be eligible, participants had to be between 18 and 40 years of age, currently enrolled in a university degree programme, healthy and free of any acute or chronic medical conditions, not taking medications known to affect sleep or cognitive performance and habitual nappers who reported taking naps at least once a week. The inclusion of habitual nappers was intended to increase the likelihood of sleep during the nap opportunity. Individuals were excluded if they had diagnosed sleep disorders, current injuries or illnesses that could limit physical activity or if they had engaged in shift work within the past 3 months. Eligibility was confirmed through a screening questionnaire, which included questions about health status, training habits and nap behaviours.

G*power software (Faul et al. 2007) was used to calculate the required sample size. Values for *α* were set at 0.05 and power at 0.80. The target effect size (Cohen's *d* = 0.38) was derived from the between‐group differences in total sleep time reported in Yoon et al. (2003), which compared total sleep time in older adults with and without a nap. Based on this effect size, the required sample size for this study was determined to be 13.

The study was conducted in accordance with the principles outlined in the Declaration of Helsinki and was approved by the local Institutional Human Research Ethics Committee. This data was collected as part of a larger study looking at the effect of sleep inertia following short and long naps on perceptual, cognitive and physical performance.

### 2.2 Experimental Design

**FIGURE 1:**
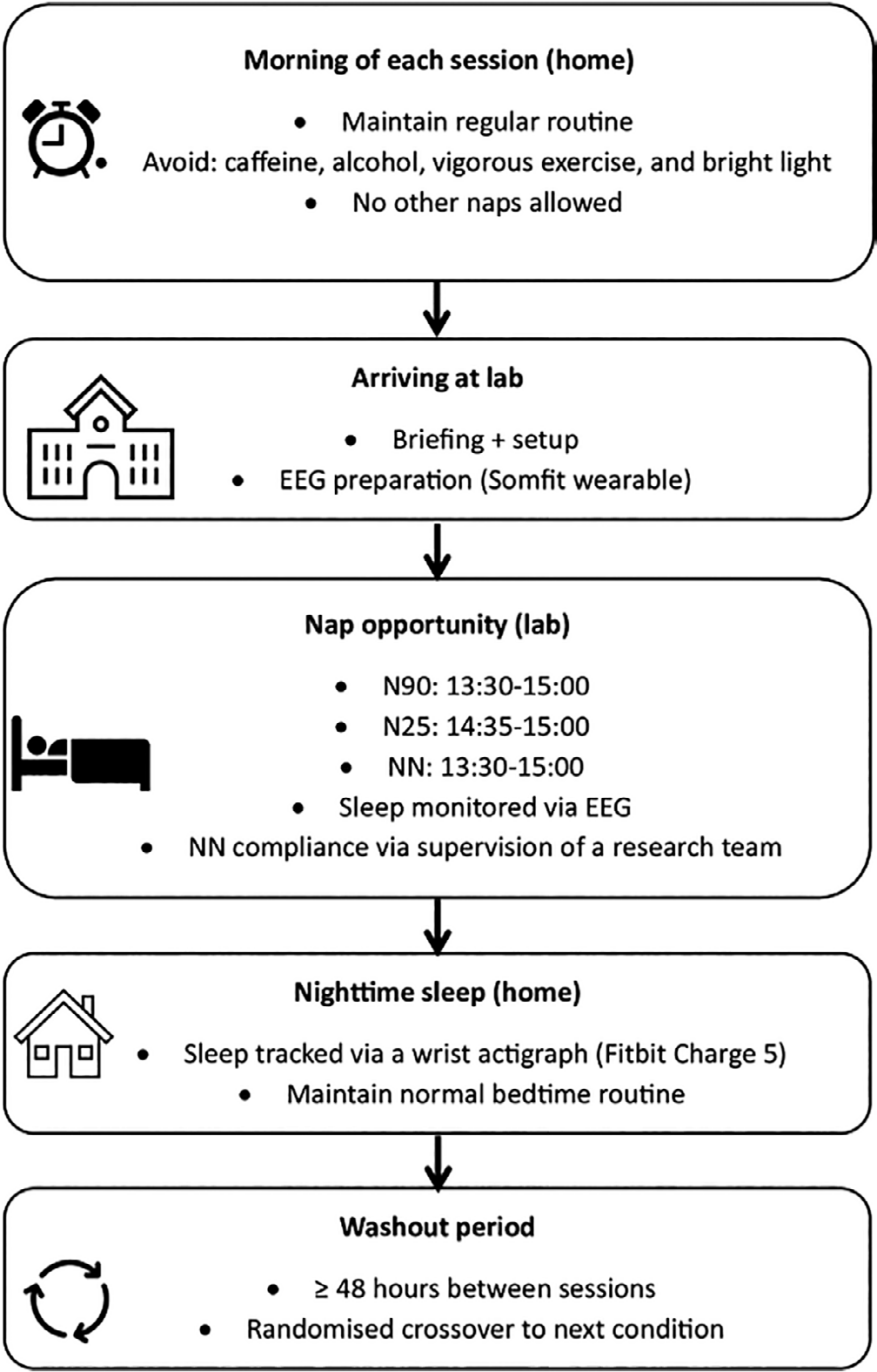
Schematic representation of the experimental design of the current study. EEG: electroencephalography; NN: NN condition; N25: 25‐min nap opportunity; N90: 90‐min nap opportunity.

This study employed a randomised, counterbalanced, crossover design with three conditions: a control condition (no nap, NN), a 25‐min nap opportunity (N25) and a 90‐min nap opportunity (N90) (Figure 1). Each condition was separated by a washout period of at least 48 h to minimise carryover effects. All participants completed the three sessions within a maximum period of 2 weeks. Participants were instructed to maintain their regular training routines and sleep–wake schedules throughout the study. On the day of each session, participants were asked to abstain from vigorous physical activity, caffeine, alcohol and other stimulants and to avoid bright light exposure before each nap opportunity and nighttime sleep assessment. They were also asked to refrain from any unscheduled naps during the experimental period. Although participants were asked to maintain consistency in their activities and lifestyle prior to each session, we did not track the specific training loads or academic demands leading up to each condition. In the control condition, participants remained in the lab, sitting quietly without napping under the supervision of a research team member to ensure compliance. For the nap conditions, participants slept in a designated sleep room equipped with a sleeping pod (Podtime, Restworks, Australia), designed to offer a comfortable and quiet environment. The room was maintained at a controlled temperature of 21°C–23°C (Caddick et al. 2018) with dim lighting (< 5 lx) to promote optimal sleep conditions. Each participant received a blanket and pillow but had the option to bring their own. The nap opportunities, conducted in the lab, were scheduled between 14:35 and 15:00 for N25 and between 13:30 and 15:00 for N90, to ensure that nap opportunities finished at the same time of day. Sleep during these naps was monitored using a wearable electroencephalography (EEG) device (see below). Following each nap session, participants wore a Fitbit Charge 5 (Fitbit Inc., San Francisco, CA, USA) at home on their wrist to track their nighttime sleep patterns.

### 2.3 Measures

The present study utilised a wearable EEG device (Somfit, Compumedics, Australia) to assess sleep metrics during the nap period. The Somfit attaches to the forehead via an adhesive patch and transmits data to the manufacturer's mobile application, compatible with both iOS and Android. The device captures EEG signals, as well as electrooculography (EOG)/electromyography (EMG) signals derived from EEG, in addition to oxygen saturation, heart rate and heart rate variability. It enables the analysis of sleep architecture and classifies sleep stages (N1, N2, N3 and REM) in accordance with the American Academy of Sleep Medicine standards. A validation study comparing Somfit to polysomnography (PSG) found an overall sleep stage agreement of 76.14% (SE: 0.79) between Somfit's automated algorithm and consensus PSG hypnograms, with a Cohen's kappa of 0.672 (SE: 0.002) (McMahon et al. 2024). Its sleep/wake classification accuracy was 89.30% (SE: 0.37), comparable to the variability observed among PSG technologists (range: 74.36%–85.50%) (McMahon et al. 2024).

The night after each nap condition, participants were asked to wear a Fitbit Charge 5 (Fitbit Inc., San Francisco, CA, USA) on their wrist to monitor sleep patterns at home. Participants could choose to wear the device on either wrist based on their personal preference (Driller, O'Donnell, et al. 2017). The Fitbit Charge 5 utilises triaxial accelerometry and Bluetooth connectivity for data synchronisation with a smartphone application. Prior research validated the accuracy of this manufacturer's actigraphy devices for measuring total sleep time and sleep onset latency in healthy adults, demonstrating comparable results to polysomnography (Haghayegh et al. 2019). Furthermore, previous studies suggested that these devices outperform many commercially available wearables and even some research‐grade actigraphy tools, making them a reliable option for assessing sleep duration in human participants (Chinoy et al. 2021). In addition, a recent validation study comparing six wearable devices to polysomnography found that the Fitbit Charge 5 showed moderate agreement for sleep stage classification (Cohen's *κ* = 0.41), supporting its use for estimating sleep architecture (Schyvens et al. 2025).

### 2.4 Statistical Analysis

Data were analysed using Statistica software (version 10, StatSoft, France).

The Shapiro–Wilk test was used to assess the normality of data distribution, while Mauchly's test was performed to evaluate the sphericity assumption for repeated‐measures factors. Parametric tests were applied when these assumptions were met. If the sphericity assumption was violated (*p* < 0.05), Greenhouse–Geisser corrections were used to adjust the degrees of freedom.

Analyses of EEG‐derived sleep metrics during nap opportunities (total sleep time, light sleep, deep sleep and REM sleep) were conducted primarily as an adherence and quality check to confirm that participants engaged in sleep as intended during each nap condition and to verify that the scheduled nap durations resulted in the expected differences in sleep obtained. EEG‐derived total sleep time was normally distributed; therefore, a paired *t* test was used to compare it between the N25 and N90 conditions. However, EEG‐derived light sleep (sleep stage N1 and N2), deep sleep (sleep stage N3) and rapid eye movement (REM, N4) sleep were not normally distributed; therefore, a Wilcoxon test was used to compare them between the N25 and N90 conditions.

Regarding actigraphy‐derived sleep metrics recorded by the Fitbit, a one‐way repeated measures ANOVA was applied to compare night's sleep following each condition (i.e., actigraphy‐derived wake time, total sleep time, time in bed, WASO, light sleep and deep sleep). When significant main effects of conditions were identified, a Bonferroni post hoc test was applied for pairwise comparisons.

Actigraphy‐derived bedtime and REM sleep recorded by the Fitbit were not normally distributed; therefore, a Friedman nonparametric analysis of variance was used. Pairwise comparisons were performed using a Wilcoxon test.

In addition to the primary one‐way repeated measures ANOVA used to assess the effect of nap condition (NN, N25, N90) on nighttime sleep parameters, exploratory analyses were conducted using analysis of covariance (ANCOVA) to control for potential confounding variables. Separate ANCOVA models were run for each nighttime sleep outcome, with EEG‐derived nap total sleep time, age and sex included as covariates. In each model, nap condition was treated as a categorical independent variable. EEG‐derived nap total sleep time and age were entered as continuous covariates, while sex was entered as a binary variable (coded as 1 = male, 0 = female). These analyses aimed to determine whether adjusting for these covariates would alter the main effects observed.

Effect sizes for variables that are normally distributed were determined using partial eta‐squared (*η*_*p*_^2^), with values of 0.01, 0.06 and 0.13 indicating *small*, *moderate* and *large* effects, respectively (Lakens 2013). For variables that were not normally distributed, effect sizes were assessed using Kendall's coefficient of concordance, which ranges from 0 (no agreement) to 1 (complete agreement) (Field 2005).

The statistical significance threshold was set at *p* < 0.05 for all analyses. Exact *p*‐values are reported, though values displayed as ‘0.000’ in statistical outputs are referred to as ‘< 0.001’.

## 3 Results

As a compliance and quality check, we confirmed that participants engaged in sleep as intended during both nap opportunities. EEG‐derived sleep metrics during both nap duration opportunities (Table 1) resulted in total sleep time and sleep stages (i.e., light sleep, deep sleep and REM sleep) being significantly higher during the N90 opportunity compared to the N25 opportunity (*p* < 0.05).

**TABLE 1:** EEG‐derived sleep metrics recorded during the 25‐min nap (N25) and the 90‐min nap (N90) opportunities.

	25‐min nap (N25)	90‐min nap (N90)	Statistic	*p*
Total sleep time (min)	10 ± 5	56 ± 26	*t* = −6.72	< 0.001
Total sleep time (%)	37%	62%	NA	NA
Light sleep—N1 and N2 (min)	9 ± 5	40 ± 17	*Z* = −6.94	< 0.001
Light sleep—N1 and N2 (%)	37%	44%	NA	NA
Deep sleep—N3 (min)	0 ± 1	9 ± 10	*Z* = 2.54	0.01
Deep sleep—N3 (%)	1%	10%	NA	NA
REM sleep—N4 (min)	0 ± 0	7 ± 11	*Z* = 2.52	0.01
REM sleep—N4 (%)	0%	8%	NA	NA

*Note*: Data shown as means ± SD. All percentages (%) represent the proportion of the total nap window (25 min for N25, 90 min for N90) spent in each stage.

Abbreviation: NA, not applicable.

**FIGURE 2:**
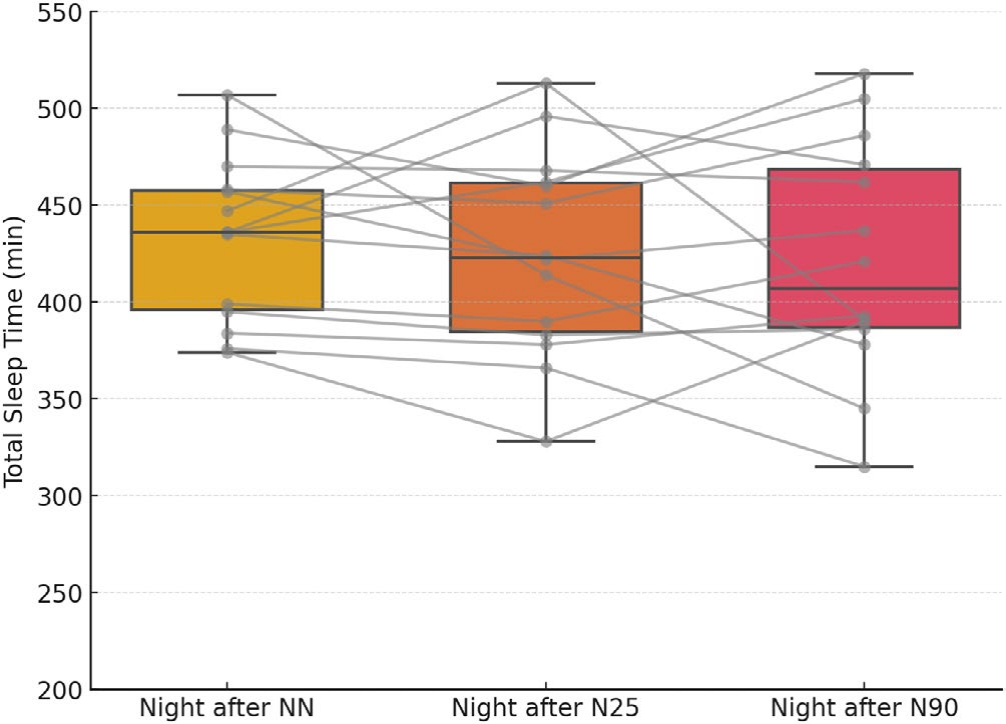
Actigraphy‐derived total sleep time (min) recorded after no nap (NN), 25‐min nap (N25) and 90‐min nap (N90) opportunities. Values are shown as box plots representing the interquartile range (IQR), with medians (horizontal line) and whiskers denoting 1.5 × IQR. Individual lines connect each participant's data across conditions to illustrate variability.

Statistical analysis revealed nonsignificant main effects of Condition on actigraphy‐derived sleep metrics recorded by the Fitbit at night after each condition, including bedtime, wake time, total sleep time (Figure 2), time in bed, WASO, light sleep, deep sleep and REM sleep. Full statistical details are presented in Table 2.

**TABLE 2:** Actigraphy‐derived characteristics of postnap night sleep (mean ± SD), including ANOVA, *p*‐value and effect size.

	Night's sleep after the control condition (NN)	Night's sleep after the 25‐min nap opportunity (N25)	Night's sleep after the 90‐min nap opportunity (N90)	ANOVA	*p*	Effect size (*η*_*p*_^2^)
Bedtime (time of day) (hh:mm)	23:01 ± 00:48	23:56 ± 00:49	23:25 ± 00:34	3.0	0.22	Kendall's *W* = 0.10
Wake time (time of day) (hh:mm)	08:29 ± 00:04	08:20 ± 00:05	07:49 ± 00:05	2.8	0.07	*η*_*p*_^2^ = 0.18
Time in bed (h:min)	8:21 ± 00:49	8:09 ± 01:05	8:06 ± 01:20	0.4	0.65	*η*_*p*_^2^ = 0.03
Total sleep time (h:min)	7:13 ± 00:42	7:05 ± 00:53	7:01 ± 01:01	0.3	0.68	*η*_*p*_^2^ = 0.02
WASO (min)	68 ± 14	63 ± 21	65 ± 26	0.2	0.76	*η*_*p*_^2^ = 0.02
Light sleep—N1 and N2 (min)	251 ± 33	245 ± 50	251 ± 51	2.4	0.29	Kendall's *W* = 0.08
Deep sleep—N3 (min)	87 ± 21	83 ± 16	80 ± 13	0.8	0.42	*η*_*p*_^2^ = 0.06
REM sleep—N4 (min)	95 ± 24	98 ± 34	91 ± 27	0.2	0.75	*η*_*p*_^2^ = 0.02

To account for potential confounding variables, additional ANCOVAs were conducted with EEG‐derived nap total sleep time, age and sex entered as covariates for each actigraphy‐derived nighttime sleep metric (Table 3). These analyses confirmed the initial findings: nap condition remained a nonsignificant predictor of all nighttime sleep outcomes, including bedtime, wake time, total sleep time, time in bed, WASO and sleep stages (*p* > 0.05). EEG‐derived nap total sleep time and age also did not significantly predict any of the nighttime sleep variables (*p* > 0.05). However, sex emerged as a significant predictor of REM sleep (*F* = 5.20, *p* = 0.03, *η*_*p*_^2^ = 0.13), with females exhibiting longer REM durations than males across conditions.

**TABLE 3:** ANCOVA results for actigraphy‐derived nighttime sleep metrics adjusting for EEG‐derived nap total sleep time, age and sex.

Outcome variable	Predictor	*F*	*p*	*η*_*p*_^2^
Bedtime	Nap condition	2.85	0.07	0.14
	EEG‐derived nap TST	1.29	0.26	0.03
	Age	1.19	0.28	0.03
	Sex	0.03	0.87	0.001
Wake time	Nap condition	0.02	0.98	0.001
	EEG‐derived nap TST	0.80	0.38	0.02
	Age	2.77	0.10	0.07
	Sex	1.19	0.28	0.03
Time in bed	Nap condition	0.47	0.63	0.03
	EEG‐derived nap TST	1.74	0.20	0.05
	Age	3.06	0.09	0.08
	Sex	2.21	0.15	0.06
Total sleep time	Nap condition	0.73	0.49	0.04
	EEG‐derived nap TST	2.84	0.10	0.07
	Age	2.08	0.16	0.05
	Sex	1.49	0.23	0.04
WASO	Nap condition	0.17	0.84	0.01
	EEG‐derived nap TST	0.01	0.92	0.000
	Age	3.28	0.08	0.08
	Sex	2.41	0.13	0.06
Light sleep	Nap condition	0.76	0.47	0.04
	EEG‐derived nap TST	1.64	0.21	0.04
	Age	3.99	0.053	0.10
	Sex	0.32	0.58	0.01
Deep sleep	Nap condition	0.88	0.42	0.05
	EEG‐derived nap TST	0.51	0.48	0.01
	Age	1.02	0.32	0.03
	Sex	2.56	0.12	0.07
REM sleep	Nap condition	0.59	0.56	0.03
	EEG‐derived nap TST	2.28	0.14	0.06
	Age	1.74	0.20	0.05
	Sex	5.20	0.03	0.13

Abbreviations: EEG = electroencephalography; Nap TST = nap total sleep time; REM = rapid eye movement; WASO = wake after sleep onset.

## 4 Discussion

Our findings show that neither a 25‐ nor 90‐min nap opportunity significantly altered key actigraphy‐derived sleep parameters (total sleep time, bedtime, WASO or sleep stage durations) compared to NN. This suggests that, within the context of this sample of student athletes, early afternoon nap opportunities, ending at 15:00, whether short or long, may not significantly disrupt nighttime sleep. However, caution is warranted in generalising beyond the tested nap opportunity durations and schedules. This is an important contribution to the literature, as it challenges the commonly held assumptions that longer nap opportunities in the afternoon could disrupt subsequent nighttime sleep, particularly, in student athletic populations.

In agreement with our results, a study conducted on collegiate athletes using subjective measures (i.e., Pittsburgh Quality Sleep Index to assess nighttime sleep and the Napping Behaviour Scale to evaluate naps) found no association between napping and poor sleep quality or duration, even when considering factors such as nap timing, frequency and length (Stephenson et al. 2022). Importantly, our use of objective measurements (i.e., EEG to monitor nap sessions and actigraphy to assess nighttime sleep) adds methodological rigour to these findings and addresses limitations of self‐reported data in prior research.

However, studies in non‐athlete populations (Campbell et al. 2005; Mograss et al. 2022; Monk et al. 2001; Ye et al. 2015) have linked napping to poorer nighttime sleep. In fact, frequent, long (> 2 h) and late (taken at 18:00–21:00) naps were linked to reduced nighttime sleep quality in college students (Ye et al. 2015), while afternoon and evening naps led to disrupted sleep in older adults (Monk et al. 2001) and young sedentary adults (Mograss et al. 2022). However, several key methodological and population differences may explain the discrepancies between our study and these previous findings. First, the nap durations in our study were considerably shorter (≤ 90 min) and all nap opportunities were scheduled to end by 15:00, ensuring they fell within the post‐lunch dip period (13:00–16:00), which is considered optimal for avoiding nighttime sleep disturbances (Boukhris et al. 2024; Lastella et al. 2021). Indeed, late naps taken closer to the biological night may suppress evening melatonin secretion and delay sleep onset (Liu et al. 2024). Therefore, the early afternoon timing of nap opportunities may have reduced the likelihood of circadian misalignment, although we did not systematically test naps at later times or assess melatonin or circadian markers. Second, while Ye et al. (2015) relied on self‐reported sleep measures, our study utilised objective sleep assessments via an EEG device to ensure nap compliance, providing more precise and reliable evaluations of sleep parameters. These methodological differences suggest that well‐timed early afternoon nap opportunities, such as those used in this study, may be less likely to interfere with nighttime sleep. However, without testing a broader range of nap times and durations, this conclusion should be interpreted cautiously. Additionally, the population differences between our study and previous studies (Campbell et al. 2005; Monk et al. 2001) could account for different results. Their studies focused on older adults, a population with different sleep physiology and typically lighter, more fragmented sleep patterns compared to young, active individuals (Campbell et al. 2005). In contrast, our participants were student athletes, involved in different sport settings and training an average of 8 ± 2 h per week. The participants of our study face higher physical demands due to training and competition, which likely increases their overall sleep need and recovery requirements compared to other populations (Boukhris et al. 2024; Lastella et al. 2021). The well‐documented benefits of physical activity on sleep (Kline et al. 2021) may further support the ability of student athletes to maintain nighttime sleep patterns, even when engaging in daytime napping opportunities. However, the interaction between training load, sleep need and nap tolerance remains unclear due to our limited assessment of training variables. These findings highlight the importance of considering individual and population‐specific factors when evaluating the impact of napping on nighttime sleep. In fact, although mean differences in actigraphy‐derived total sleep time between conditions were not statistically significant, visual inspection of Figure 2 revealed some differences in variability across nap conditions, as reflected by the differing box lengths and error bars. This variability suggests that individual responses to napping may differ, even if the overall means are not statistically distinct. Some participants may have experienced increases or decreases in nighttime sleep following napping, depending on their baseline sleep need, circadian preference or sensitivity to daytime sleep.

A potential explanation for the absence of significant nighttime sleep disruptions in our study is that our participants were habitual nappers, whose sleep–wake cycles have adapted to include naps. This may not apply to nonhabitual nappers, who could be more susceptible to sleep homeostasis disruption. Additionally, our cohort did not consistently reach the higher sleep durations recommended for athletes (9–10 h), possibly reducing the risk of napping interfering with nighttime sleep (Mah et al. 2011).

While our findings suggest that afternoon nap opportunities ending by 15:00 do not disrupt nighttime sleep in habitual napping student‐athletes, it is important to avoid making broad recommendations without accounting for individual differences. Napping may benefit those with high training loads, early training or academic demands (Mesas et al. 2023; Takahashi 2003; Vidal et al. 2025), but may worsen sleep in those with insomnia, sleep disorders or sensitivity to circadian disruption (Jang et al. 2018; Takahashi 2003). Optimal nap duration and timing likely vary depending on individual sleep needs, chronotype, academic workload and lifestyle demands (Lastella et al. 2021). As such, nap recommendations should be personalised, weighing potential benefits against risks in light of the athlete's unique sleep patterns, training demands and academic responsibilities. Future research should identify which subgroups benefit most or least from napping.

While the N90 allowed participants to accumulate more light sleep (~40 min), deep sleep (~9 min) and REM sleep (~7 min) compared to the N25 (~9 min of light sleep, 0 min of deep sleep and 0 min of REM sleep), these differences did not translate into significant changes in nighttime sleep, suggesting that additional daytime sleep stages did not interfere with nocturnal sleep cycles. The relatively short deep and REM sleep durations during N90 may not have been enough to affect nighttime sleep architecture.

Exploratory ANCOVAs, controlling for EEG‐derived nap total sleep time, age and sex, supported our main findings. Specifically, the main effects of nap condition on nighttime sleep outcomes remained nonsignificant after adjusting for these covariates. This suggests that neither nap total sleep time nor condition predicted nighttime sleep outcomes, possibly due to short nap durations or timing relative to circadian phases. However, sex was a significant predictor of REM sleep, with females showing longer REM periods, aligning with prior evidence of sex differences in sleep architecture, particularly in REM sleep, which may reflect hormonal or neurobiological factors (Mong and Cusmano 2016). Future studies should further explore sex‐specific effects and whether longer naps or different timings affect sleep homeostasis.

## 5 Limitations

This study is not without limitations. The generalisability of our findings to other athlete populations (e.g., elite athletes) or non‐athletes warrants further investigation. Although individuals with known sleep disorders were excluded, habitual napping in student athletes may still reflect subclinical sleep disturbances, potentially due to early training schedules or academic demands (Mah et al. 2018). This limits the generalisability to nonhabitual nappers or those with different lifestyles. Our participants, classified as Tier 2 trained/developmental athletes (~2 h of training, four times per week), may not experience the same recovery needs as higher‐tier athletes (McKay et al. 2022), warranting caution when generalising these results. To better understand the influence of training status, replication of this protocol in more highly trained or elite cohorts is recommended. While participants were required to report napping at least once per week to be eligible, we did not quantify their habitual nap frequency, duration, timing, chronotype or sleep–wake patterns before the study, which may have influenced individual responses to the nap conditions (Boukhris et al. 2024; Lastella et al. 2021; Mesas et al. 2023). Future research should consider assessing habitual napping patterns with prospective sleep diaries or actigraphy in the week(s) prior to testing. Although nap opportunities ended at the same time (15:00) across conditions, fixing nap duration by clock time may have introduced variability in sleep architecture due to inter‐individual circadian differences (Åkerstedt and Gillberg 1981). Future studies could reduce this circadian variability by assigning nap opportunities relative to individual sleep–wake schedules (e.g., hours post‐wake) or circadian phase (e.g., hours before melatonin onset). Additionally, sessions were conducted on both weekdays and weekends, possibly affecting prior night sleep and daytime routines. Future studies should standardise testing days or at least monitor and control for these contextual factors. Although the Fitbit Charge 5 shows moderate agreement with polysomnography for sleep staging (Schyvens et al. 2025), it remains less accurate than gold‐standard measures. Complementing wearable‐derived data with full polysomnography assessments could strengthen future investigations. Finally, as this study only examined the acute effects of a single nap, future research should explore the long‐term impact of regular napping on sleep patterns in athletes. Longitudinal or real‐world observational studies could provide further insight into how repeated daytime naps influence nightly sleep, recovery and overall performance across varying training and academic demands.

## 6 Conclusion

In trained/developmental student athletes, afternoon naps ending by 15:00, even with an opportunity of 90‐min duration, do not negatively impact subsequent nighttime sleep. Therefore, strategically implemented naps could be a valuable recovery tool without compromising nighttime sleep quality. Future research should explore whether personalised nap recommendations, considering individual differences in sleep need, chronotype, training demands, academic load and sleep health status, are more effective in optimising recovery and sleep outcomes.

## Author Contributions

**Omar Boukhris:** conceptualization, investigation, writing – original draft, methodology, writing – review and editing, software, formal analysis, data curation. **Haresh Suppiah:** conceptualization, investigation, methodology, writing – review and editing, supervision, data curation. **Matthew Driller:** conceptualization, investigation, methodology, writing – review and editing, supervision, data curation.

## Ethics Statement

The present study was approved by the Human Research Committee at La Trobe University.

## Conflicts of Interest

The authors declare no conflicts of interest.

## Data Availability

The data that support the findings of this study are available on request from the corresponding author. The data are not publicly available due to privacy or ethical restrictions.
